# High Electrochemical Performance of Bi_2_WO_6_/Carbon Nano-Onion Composites as Electrode Materials for Pseudocapacitors

**DOI:** 10.3389/fchem.2020.00577

**Published:** 2020-07-31

**Authors:** Weike Zhang, Lin Peng, Jiawei Wang, Chunli Guo, Siew Hwa Chan, Lan Zhang

**Affiliations:** ^1^Institute of New Carbon Materials, Taiyuan University of Technology, Taiyuan, China; ^2^Beijing Huaxin Zhiyuan Taiyuan Branch, Taiyuan, China; ^3^School of Chemical Engineering and Technology, Tianjin University, Tianjin, China; ^4^School of Material Science and Engineering, Taiyuan University of Technology, Taiyuan, China; ^5^Energy Research Institute at NTU (ERIAN), Nanyang Technological University, Singapore, Singapore

**Keywords:** Bi_2_WO_6_, carbon nano-onions, pseudocapacitors, electrochemical, electrode materials

## Abstract

Bi_2_WO_6_/CNO (CNO, carbon nano-onion) composites are synthesized via a facile low-cost hydrothermal method and are used pseudocapacitor electrode material. X-ray diffraction (XRD), Fourier transform infrared spectra (FT-IR), scanning electron microscopy (SEM), transmission electron microscopy (TEM), N_2_ adsorption-desorption techniques, and X-ray photoelectron spectroscopy (XPS) measurements are used to characterize the synthesized composite powders. The electrochemical performances of the composite electrodes are studied by cycle voltammetry, charge-discharge, and electrochemical impedance spectroscopy. The results indicate that the specific capacitance of the Bi_2_WO_6_/CNO composite materials reaches up to 640.2 F/g at a current density of 3 mA/cm^2^ and higher than that of pristine Bi_2_WO_6_, 359.1 F/g. The capability of the prepared pseudocapacitor remains 90.15% after 1,000 cycles of charge-discharge cycling measurement. The cell performance and stability can be enhanced by further optimization and modification of the composition and microstructure of the electrode of the cell.

## Introduction

According to the BP Statistical Review of World Energy 2018, renewable power (solar, wind, tide, etc.) hit a new high of 3.6% in that year. The limitation of renewable energy is its intermittence. Thus, the market desires new energy storage technology to store such intermittent energy in large quantities. Furthermore, with the development of new energy vehicles, the market demand for high energy density capacitors has also increased accordingly. Thus, supercapacitors have attracted a lot of attention due to their higher energy and power density as compared with traditional capacitors and batteries and are also well-known as electrochemical capacitors (Bolloju et al., 2019). Supercapacitors are classified into two types, pseudocapacitors and electric double-layer capacitors (EDLC), according to the energy storage mechanism. The success of EDLC is due to the charge being accumulated on the electrode under the function of a physical electric field, which can achieve the aim of separation of charges from the electrode–electrolyte interface, and the capacitance of EDLC using carbon materials as the electrode gives a higher specific capacitance compared with other materials (Xie et al., 2019). However, the specific capacitance has not yet met the requirement for application.

The charge storage of pseudocapacitors involves Faradaic reactions at the surface of electrode materials, which are achieved by redox reactions, intercalation, and electrosorption in metal oxides and conductive polymers. Compared with EDLC, pseudocapacitors using metal oxide as the electrode, which could obtain higher specific capacitance, have attracted more attention. Numerous works have been published regarding using metal oxides, such as RuO_2_ (Arunachalam et al., 2019), MnO_2_ (Wu et al., 2018), NiO (Saravanakumar et al., 2018) and Co_3_O_4_ (Lai et al., 2018), etc., as electrodes for pseudocapacitors. Unfortunately, it is still difficult to meet the pseudocapacitance requirement for application because of the poor conductivity and low specific surface area of metal oxide.

Bismuth tungstate (Bi_2_WO_6_), which is one of the Aurivillius oxides, possesses a layered structure constructed by the alternation of perovskite-like units (WO_4_)^2−^ and (Bi_2_O_2_)^2+^ layers (Hojamberdiev et al., 2014) and has received a large amount of interest because of its non-toxic, strong oxidized characteristics and gas sensitivity to alcohol (Lee et al., 2012; Phu et al., 2015; Huang et al., 2017). In addition, Bi_2_WO_6_ materials have also been used as visible-light-driven photocatalysts to degrade pollutants in water (Yang Y. et al., 2018) and as electrode materials for Li-ion batteries (Wu et al., 2007), etc. Furthermore, Nithya et al. (2013) demonstrated that Bi_2_WO_6_ is a promising electrode material for pseudocapacitors and that, while the specific capacitance of pristine Bi_2_WO_6_ was 304 F/g at 3 mA/cm^2^, only 47% of the specific capacitance was maintained after 500 charge-discharge cycles; thus, capacitance retention should be improved. In addition, it should be noted that the electrochemical reaction of pseudocapacitors only occurred on the surface of electrode materials. The performance of the pseudocapacitance can be further improved by increasing the surface area of electrode materials.

Until now, many methods have been reported for improving the electrochemical performance, such as synthesizing metal oxide/carbon (graphene, carbon nanotubes, active carbon, etc.) binary composites (Yu et al., 2015), surfactant/polymer-assisted synthesis (Choi et al., 2008) and preparation of complex heterostructured nanomaterials (Jiao et al., 2014). Among these, preparing carbon-based binary composite materials is a simple and effective method of improving electrochemical performance, since carbon materials provide a pathway that is a benefit for the transfer of charges and the acceleration of redox reactions (Shahid et al., 2012). Carbon nano-onions (CNOs), which are one of the members of the carbon family, were first discovered by Iijima (1980) through electron beam irradiation of carbon soot in 1980. The as-synthesized 0-D CNOs produced via the chemical vapor deposition (CVD) method show excellent dispersibility and moderate diameter (5–80 nm). Pech et al. (2010) proved that, compared with CNT and carbon black, CNOs have low electrical resistance because of the open-surface system, which contributed to the migration of ions (Tian et al., 2020). Overall, these characteristics of CNOs are favorable for electrochemical application.

Herein, in order to make full use of the low electrical resistance of CNOs and the excellent pseudocapacitive properties of Bi_2_WO_6_, composites of Bi_2_WO_6_/CNOs were prepared via a simple hydrothermal method and were characterized using different techniques: XRD, FTIR, TEM, and so on. Furthermore, the electrochemical performances of the composites were studied.

## Experimental

### Preparation

The reagents used were of analytical grade. The CNOs used in this experimental study were obtained from Shanxi Zhongxing Environmental and Energy Technology Co., Ltd.

Bi_2_WO_6_/CNOs were prepared via a hydrothermal method. Briefly, 8 mmol Bi(NO_3_)_3_·5H_2_O was dissolved in 20 mL ethylene glycol and marked as solution A, while 4 mmol Na_2_WO_4_ and a certain amount of CNO (2wt.%, 6wt.%, and 10 wt.%) powders were added to 20 mL distilled water and marked as solution B. Next, solution B was added into solution A by the droplet, and the pH value of the mixture was adjusted by diluted NaOH solution to between 7 and 8. Subsequently, the precursor was poured into a 100-mL autoclave with Teflon-lined stainless steel and heated at 160°C for 18 h in an oven. After cooling down the system naturally, the solid-state deposit was collected and washed with distilled water and absolute ethanol several times. Finally, the as-prepared products were dried at 80°C. For a comparative study, the pure Bi_2_WO_6_ was also prepared under the same conditions but without the CNOs.

In this paper, for simplification purpose, the as-synthesized Bi_2_WO_6_/CNO composite materials with different proportions of CNOs are denoted as BWO/xCNOs (*x* = 0, 2, 6, 10), respectively.

### Characterization

The crystal phase of BWO, CNOs, and BWO/CNOs were characterized using an XRD (DX-2700X, China) with monochromatized Cu-Kα radiation (λ = 1.5406Å) at room temperature. The FT-IR of these three kinds of samples were collected on a Thermo Nicolet iS10 spectrophotometer (USA). The morphology and microstructures of samples were investigated by SEM (JEM-6700) and TEM (JEM-2100f). The Brunauer-Emmett-Teller (BET) method and the Barrett-Joyner—Halenda (BJH) equation (Quadrasorb SI, USA) were used to investigate the surface areas and porosity of the samples. The chemical states of elements of samples were explored by XPS (Amicus Budget, Japan).

The electrochemical performance of the electrodes, respectively, made with BWO and BWO/CNOs was tested on a CHI 760E workstation with a conventional three-electrode setup using Pt wire as the counter electrode and saturated calomel electrode (SCE) as the reference electrode. The working electrode was prepared from active materials as follows: BWO/xCNOs, carbon black, and polyvinylidene fluoride (PVDF) were mixed together by using N-methyl 2 pyrrolidone as a solvent at a weight ratio of 8: 1: 1 to form a slurry. The slurry was then uniformly coated onto Ni foam over an area of 1 cm^2^ and dried at 80°C overnight. The loaded mass of the active materials on the Ni foam was obtained by measurement of the weight of the specimen before and after the coating treatment. The loading of active materials was 1 mg/cm^2^, taking the average value over 20 specimens. The electrolyte used was 1 M KOH aqueous solution. Cyclic voltammetry (CV), galvanostatic charge-discharge (GCD), and electrochemical impedance spectroscopy (EIS) measurements were used to investigate the electrochemical performance of samples.

## Results and Discussion

### Characterization of the Samples

**Figure 1:**
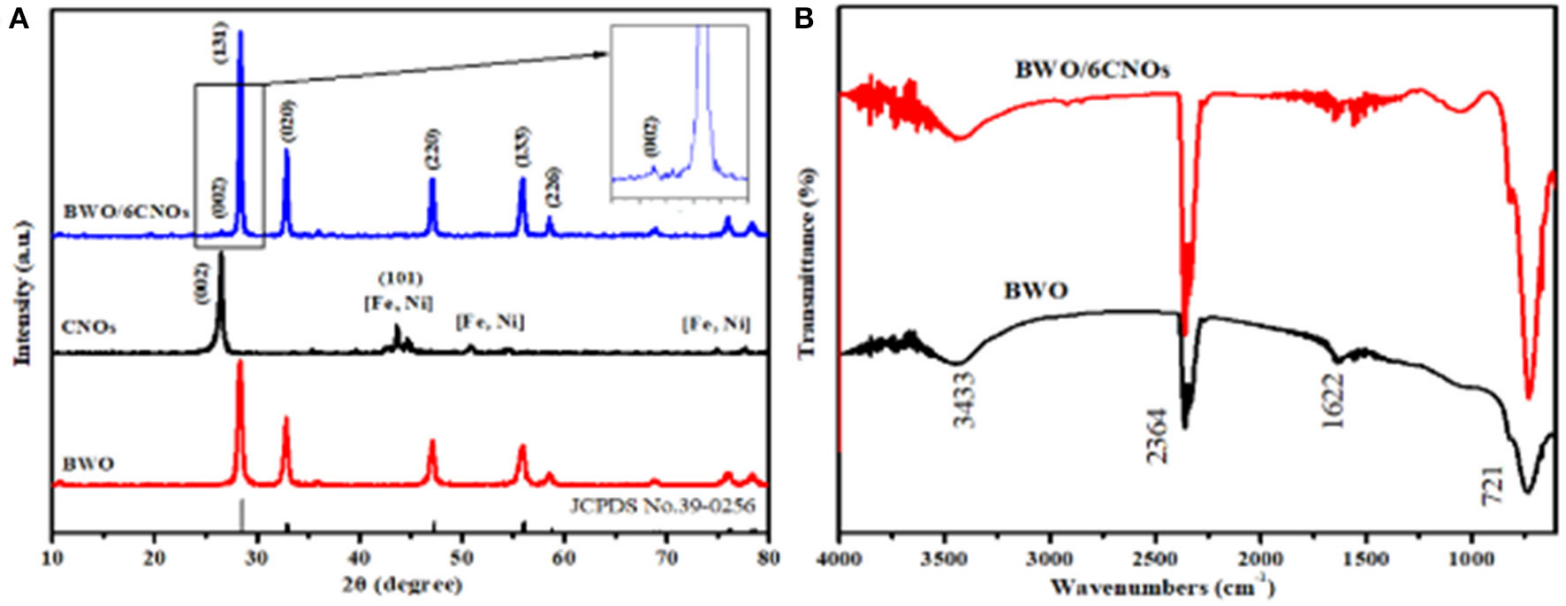
XRD patterns **(A)** of CNOs, BWO, and BWO/6CNOs and FT-IR spectra **(B)** of BWO and BWO/6CNOs.

The crystal structure of CNO, BWO, and BWO/6CNO samples were investigated by XRD, and the results are shown in Figure 1A. Referring to JCPDS Card No. 39-0256 in the studied 2θ range from 10° to 80°, it is obvious that the five main diffraction peaks of BWO located at 28.4°, 33.0°, 47.4°, 56.1°, and 58.8° correspond to the (131), (020), (220), (133), and (226) crystalline planes of BWO, respectively (Zhao et al., 2018). For the CNO particles, a significant diffraction reflection found at 26.6° is attributed to the graphite lattice plane of (002), which gives evidence for the high graphitization of the CNOs. The crystalline phases of BWO and CNOs were detected in the prepared composite materials, which means the BWO/6CNOs had been successfully synthesized by this low-cost hydrothermal method and that the phase formation of BWO had not been affected by CNOs.

To further investigate the chemical structure of the samples, the FT-IR spectra of pure BWO and BWO/6CNOs are shown in Figure 1B. The broad adsorption bands at 3,433 cm^−1^ and 1,622 cm^−1^ corresponded to O-H stretching vibrations (Bo et al., 2009). The Bi-O, W-O, and W-O-W bridging stretching modes of the samples were observed in the range of 400–1,000 cm^−1^. Furthermore, the FT-IR spectrum of BWO/6CNOs is similar to that of BWO, indicating BWO as the main component of the composites.

**Figure 2:**
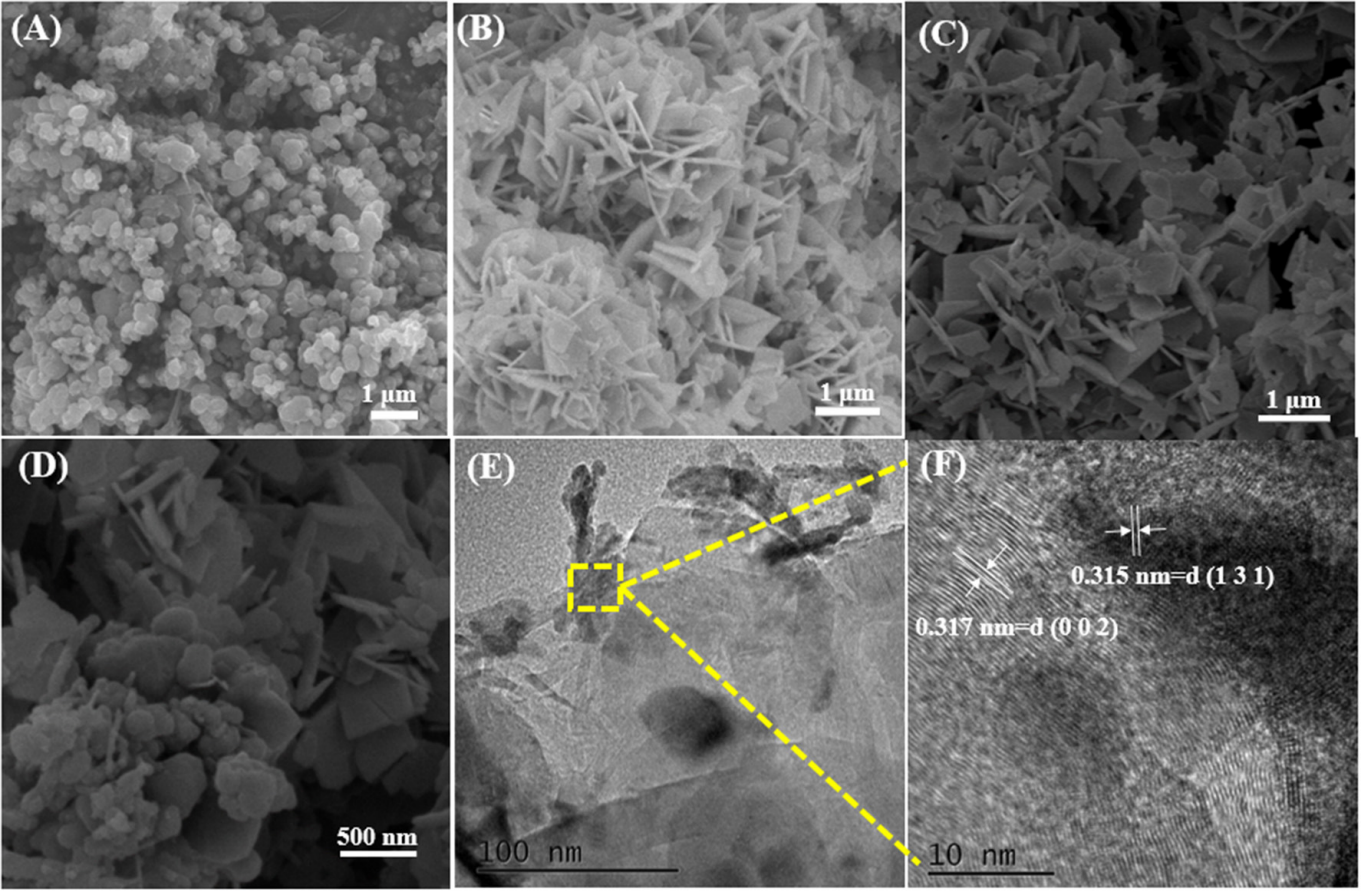
SEM images of CNOs **(A)**, BWO **(B)**, and BWO/6CNOs **(C,D)**, and TEM and HRTEM images of BWO/6CNOs **(E,F)**.

The morphology and microstructure of CNOs, BWO, and BWO/6CNO composite were explored by examining the SEM and TEM images. As shown in Figure 2A, the particle sizes of CNOs are in the range of 5–80 nm. The pure BWO presents a plate structure (Figure 2B). After the CNOs are added to BWO, the plate thickness increases obviously (Figures 2C,D), and the surface presents rough and irregular features. The close contact between BWO and CNOs makes the charge storage increase significantly. Moreover, the CNOs act like bridges between BWO nanoflakes, which facilitates the transmission of electrons and further promotes the redox reaction in supercapacitors.

TEM images of BWO/6CNO composite are also presented in Figures 2C,D. The lattice spacing was about 0.315 nm and 0.317 nm, respectively, well-matching the (131) plane of BWO and (200) plane of CNOs (Figures 2E,F), respectively. CNOs were connected with BWO closely, which is consistent with the SEM images and prove heterojunction formation between BWO and CNOs.

**Figure 3:**
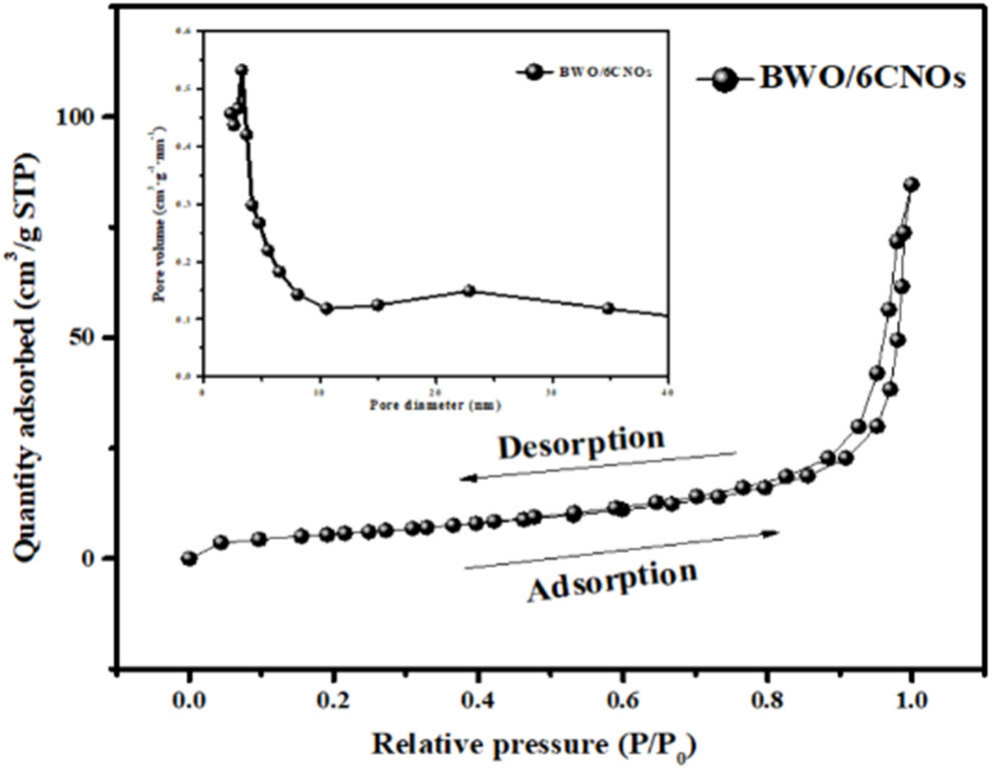
The N_2_ adsorption-desorption isotherms of as-prepared BWO/6CNOs.

BET measurement was used to analyze the specific surface area and porosity of BWO/6CNOs, and the N_2_ adsorption/desorption isotherms of the sample are shown in Figure 3. The sample showed type VI isotherms with reversible characteristics accompanied by H3 hysteresis loops. This feature indicated a mesoporous structure. Furthermore, the BET surface area and pore volume of BWO/6CNO composite were 18.099 m^2^/g and 0.063 cm^3^/g, respectively. The specific surface area of BWO and CNOs are 103.441 m^2^/g and 14.13 m^2^/g, and the decreased BET surface area of BWO/CNOs is mainly due to blocking of active sites by CNOs on the surface of BWO. The dominant pore size was distributed around 3.267 nm. The high surface area and well-dispersed pore structure of the BWO/6CNO composite are promising for enhancing the transportation and diffusion of electrolyte ions during the charge/discharge process.

**Figure 4:**
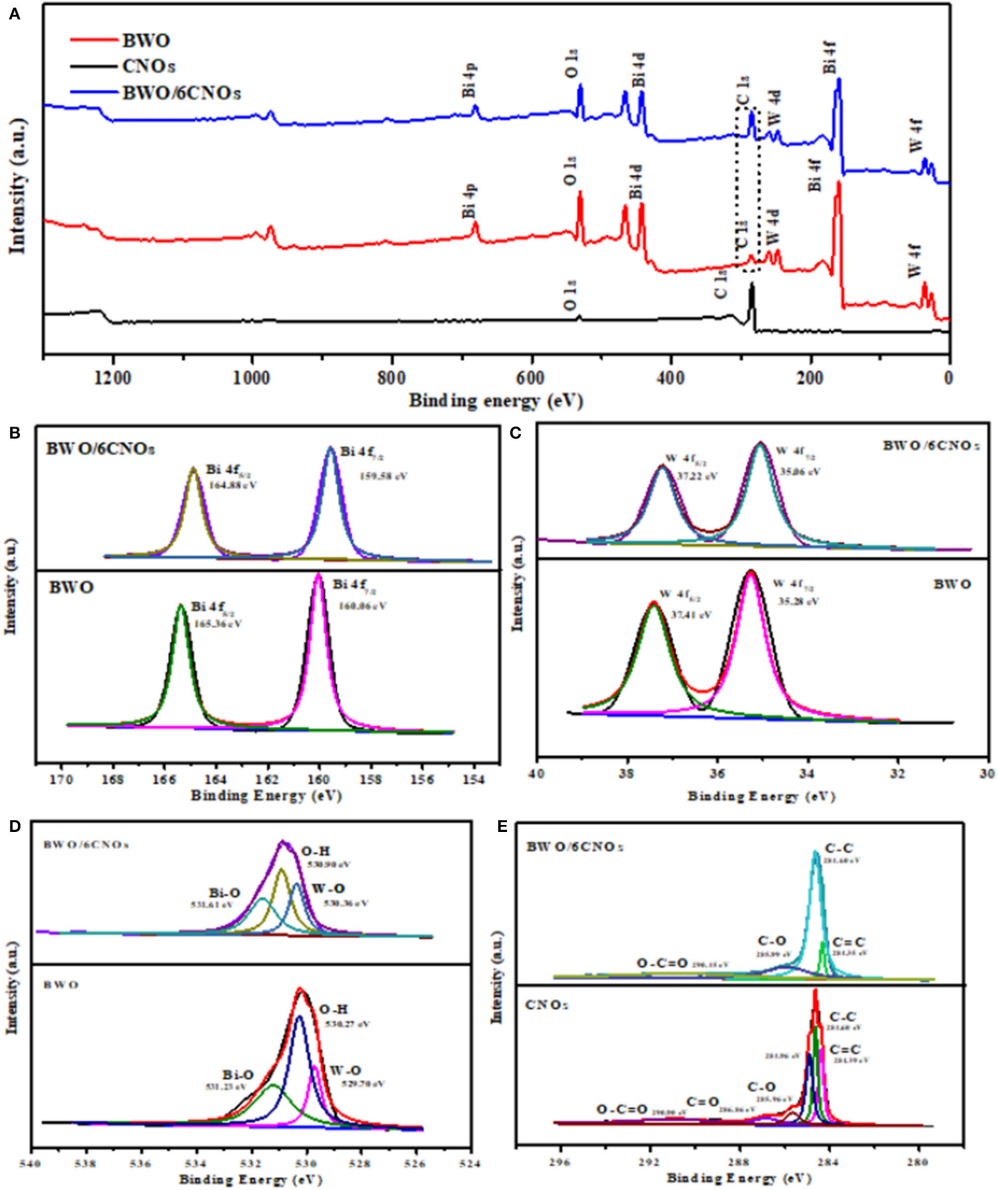
XPS survey spectra of CNOs, BWO, and BWO/6CNOs **(A)** and high-resolution spectra of Bi 4f **(B)**, W 4f **(C)**, O 1s **(D)**, and C 1s **(E)** in BWO, CNOs, and BWO/6CNOs.

The bonding status and elemental composition of the sample surface were studied by XPS. The full survey spectra in Figure 4A show that the elements Bi, O, W, and C existed in BWO and BWO/6CNOs. The C element in pure BWO was due to the residual carbon from the solution or the hydrocarbon from instrument (Wang et al., 2012). The peaks marked at 160.06 and 165.36 eV correspond to Bi 4f_5/2_ and Bi 4f_7/2_ (Figure 4B), which are orbits of the Bi^3+^ oxidation state (Ma D. et al., 2016; Wang et al., 2019a). The peaks with binding energy at 35.28 and 37.41 eV were W 4f_5/2_ and W 4f_7/2_ (Figure 4C), respectively, indicating the existence of W^6+^ (Xia et al., 2014). Notably, the spectra of O 1s can be divided into three peaks at 529.70 eV, 530.27 eV, and 531.23 eV, which reflected the different O states in W-O (Dong et al., 2017), O-H (Guo et al., 2012) and Bi-O (Li et al., 2012) (Figure 4D). In the case of BWO/6CNOs, the binding energy of Bi 4f, W 4f, and O 1s were shifted prominently to lower binding energies, which confirmed the existence of a certain chemical interaction between BWO and CNOs. As shown in Figure 4E, the C-C bond was observed at 284.60 eV, and the peak located at 284.96 eV is consistent with the sp^2^-hybrid mode on the surface of CNOs. Besides, the characteristic peaks of CNOs for C=C, C-O, C=O, and O-C=O were located at 284.39, 285.96, 286.86, and 290.90 eV, respectively. The C 1s spectra of BWO/6CNOs contained only four peaks, viz., 284.35, 284.60, 285.99, and 290.45 eV, which were related to C=C, C-C, C=O, and O-C=O bonds (An et al., 2007 and Wang et al., 2019b). The proportion of CNOs in the composite materials was <10 wt.%; thus, some peaks of C 1s with low intensity were not detected.

### Electrochemical Analysis

**Figure 5:**
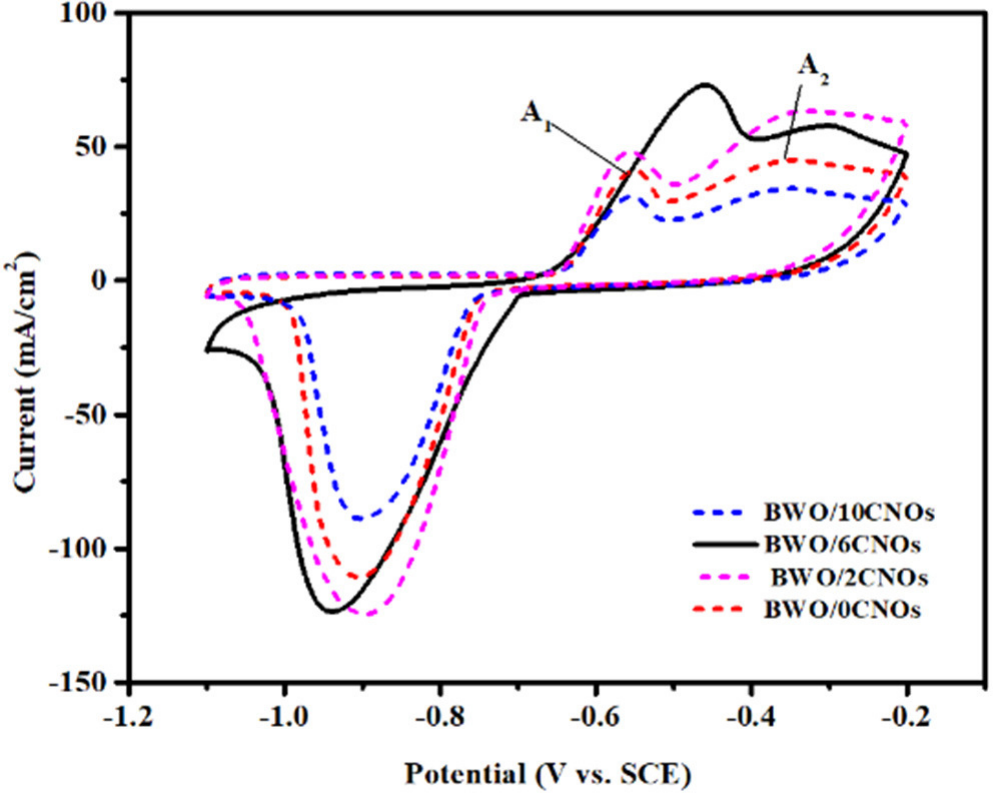
CV curves of composites with different proportions CNO additives at a scan rate of 30 mV/s.

CV is an important technique that gives valuable information about electrochemical processes. Figure 5 displays the CV curves of BWO/6CNOs as a function of CNOs at the scan rate of 30 mV/s in 1 M KOH electrolyte. The potential windows studied are between −1.1V−0.2V because the working electrode shows superior performance in this condition while it undergoes vigorous reaction at other voltages (Zhang et al., 2016). As shown in Figure 5, a single reduction peak and two oxidation peaks were obviously found for the samples; these were caused by the oxidation and reduction reactions occurring on the surface of the sample. Besides, it is remarkable that the CV curves of BWO/6CNOs are comparable to the CV curves of Bi_2_O_3_, BiPO_4_, BiVO_4_, and CuBi_2_O_4_ (Vivier et al., 2001; Khan et al., 2014; Nithya et al., 2015), which belong to the bismuth-based oxides. The result suggests that the state of bismuth makes a great difference in the electrochemical process. According to the redox details provided by Vivier et al. (2000), the redox peaks were related to the faradic processes of Bi(III) ↔ Bi (0). The reduction peak observed at −0.9V was attributable to the reduction process of Bi (III) to Bi (0), and the pathway can be described by Equations 1–4, while the relative oxidation peaks of Bi (0) to Bi (III) were located at −0.57V (A_1_) and −0.41V (A_2_), which was related to the process described in Equations 5–7.

(1) $$\begin{array}{r} \text{Bi}{\text{O}}_{2\;\left(\text{ads}\right)}^{-}\;+\;{\text{e}}^{-}\;\to \text{ Bi}{\text{O}}_{2\;\left(\text{ads}\right)}^{2-} \end{array}$$

(2) $$\begin{array}{l} 2{\text{H}}_{2}\text{O }+\;3{\text{BiO}}_{2}^{2-}\;\overset{\text{Disproportionation}}{↔}\;2{\text{BiO}}_{2}^{2-}\;+\;4{\text{OH}}^{-}\\ \;+{\text{Bi}}^{\left(\text{0}\right)} \end{array}$$

(3) $$\begin{array}{r} \text{B}{\text{i}}^{\left(0\right)}\;\to \text{ Bi}{\;}_{\left(\text{metal}\right)} \end{array}$$

(4) $$\begin{array}{r} \text{B}{\text{i}}_{\;\left(\text{metal}\right)}\;\to \text{ B}{\text{i}}^{+}\;+\;{\text{e}}^{-} \end{array}$$

(5) $$\begin{array}{r} 3{\text{Bi}}^{+}\;\overset{\text{Disproportionation}}{↔}\;{\text{Bi}}^{3+}\;+\;2{\text{Bi}}_{\left(\text{metal}\right)} \end{array}$$

(6) $$\begin{array}{r} 3\text{O}{\text{H}}^{-}\;+\text{ B}{\text{i}}^{3+}\;\to \text{ Bi}{\left(\text{OH}\right)}_{3} \end{array}$$

(7) $$\begin{array}{r} \text{Bi}{\left(\text{OH}\right)}_{3}\;\to \text{ BiOOH }+\;{\text{H}}_{2}\text{O} \end{array}$$

To further investigate the electrochemical performance of the samples with different proportions of CNO additives, specific capacitance is calculated using the following equation according to Figure 5:

(8) $$\begin{array}{r} C_{s}\;=\;\frac{\int I\text{dV}}{2\;\times \;\Delta V\;\times \;m\;\times \;\vartheta } \end{array}$$

where C_s_ is the specific capacitance (F/g) in CV. ∫*IdV* represents the CV integrated current area of the samples. ΔV, m, and ϑ are the potential window (V), the mass of active materials (g), and the scan rate, respectively.

It can be seen that the specific capacitance of samples mainly depend on the area of CV curves, and the specific capacitance of BWO/6CNOs is calculated to be 833.3 F/g, which is larger than that of the other composites (the specific capacitances of BWO/0CNOs, BWO/2CNOs, and BWO/10CNOs are 611.1, 825, and 487 F/g, respectively). The improvement of specific capacitance up to BWO/6CNOs is due to the superior electron-mobility of CNOs. While the electrochemical performances were not enhanced as the content of CNOs increased, this is because excessive CNOs could be a combination center rather than an electron pathway, causing a high recombination rate in MCNOs, which would reduce the electrochemical performance (Zhu et al., 2018). In summary, the BWO/6CNO composite shows the best electrochemical performance within the scope of our study, and further improvements will be reported in the future. Thus, the BWO/6CNO composite was used in the subsequent experiments to further explore its electrochemical performance.

**Figure 6:**
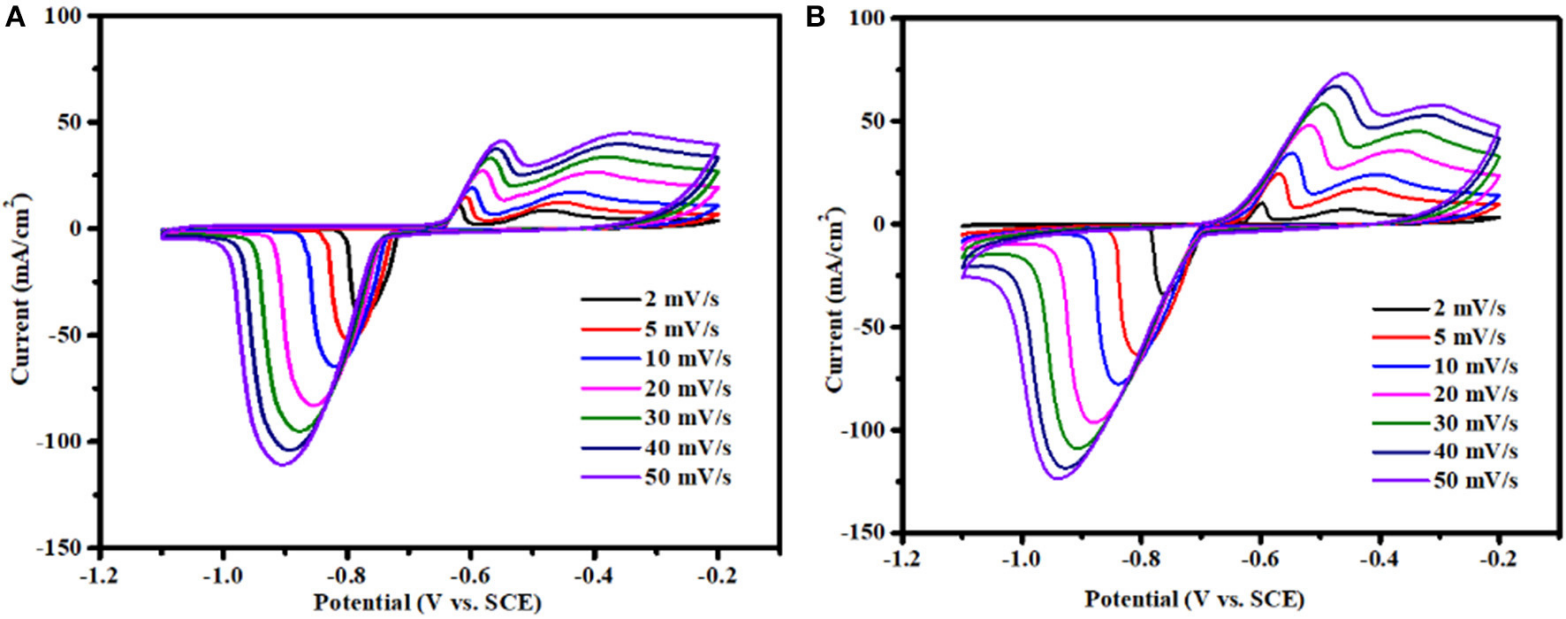
**(A)** CV curves of BWO and **(B)** BWO/6CNOs at different scan rates.

The electrochemical performances of BWO and BWO/6CNO samples were also evaluated by CV testing in a potential window from −1.1 V to −0.2 V as a function of scan rates from 2 to 50 mV/s in 1 M KOH electrolyte, and the results are presented in Figures 6A,B. The CV integrated current areas of BWO and BWO/6CNOs are decreased with an increase in the scan rate, which is attributed to a decrease in active sites involved in redox reactions (Nithya et al., 2013). That is to say, the ions have enough time to move to the surface of the electrode at low scan rates and make full use of the active materials, on the contrary, only the process of surface adsorption takes place at high scan rates because the ions do not have enough time to utilize the materials (Selvan et al., 2008). It is found that with an increase in scanning rate, a slight shift appears in the curves, which is assigned to the drop in internal resistance.

**Figure 7:**
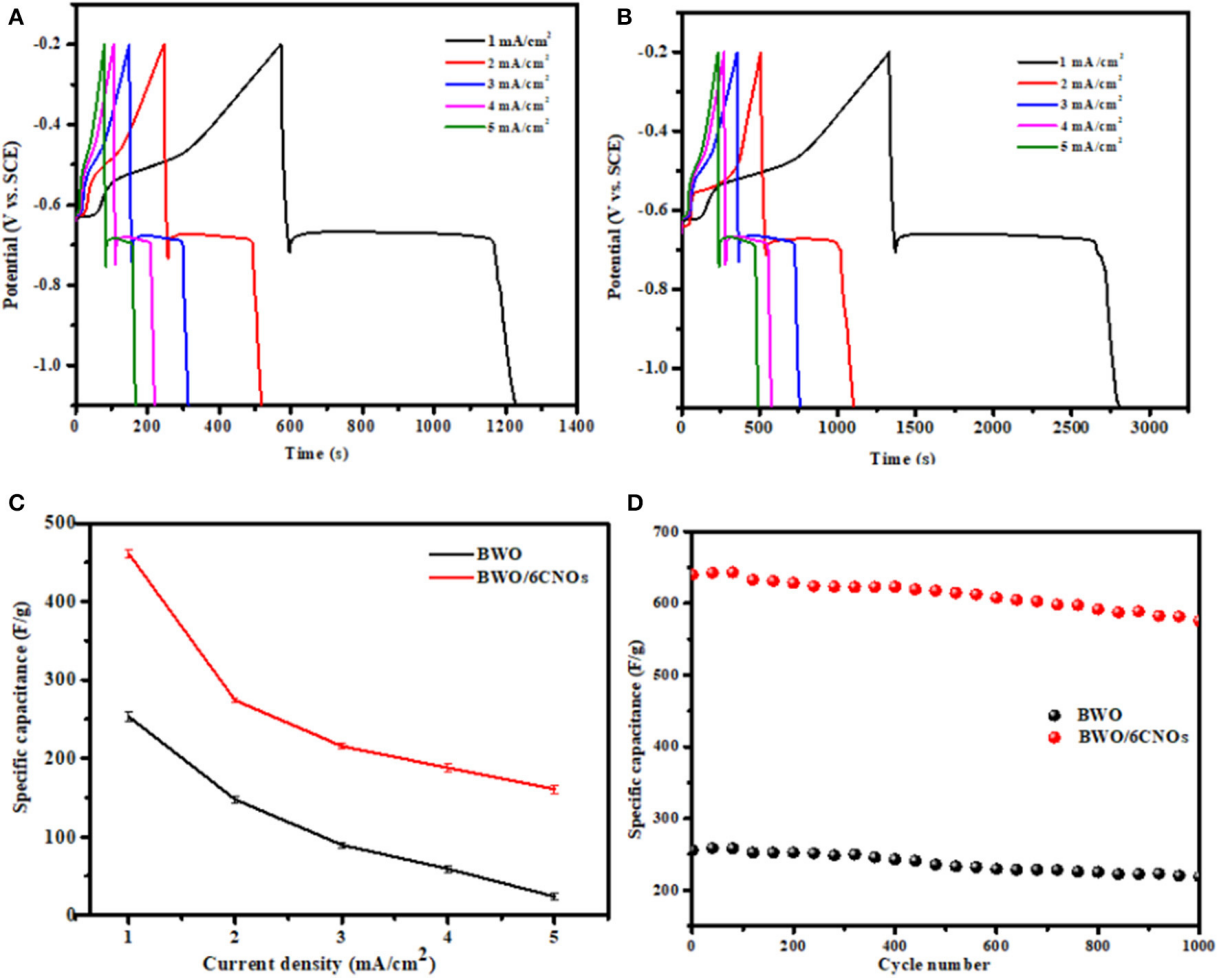
**(A)** GCD curves of BWO and **(B)** BWO/6CNOs at different current densities. **(C)** Specific capacitance of BWO and BWO/6CNOs at various current densities. **(D)** Capacitance retention of BWO/6CNOs at a current density of 3 mA/cm^2^ for 1,000 cycles.

To further investigate the electrochemical performance of the samples, GCD curves were measured; the results are shown in Figure 7. It can be seen that the curves of BWO and BWO/6CNOs show the same non-linear characteristic, which is because of the redox mechanism operating during the electrochemical reaction, and the result is consistent with the CV results presented in Figure 6. The typical discharge curves of pseudocapacitors consist of three stages, i.e., an initial sudden drop owing to the solution resistance, followed by a plateau ascribed to the electrochemical reaction, and a final decrease arising from the ion concentration polarization (Mo and Brodd, 2005).

As shown in Figures 7A,B, it is obvious that a higher discharge time is found for the BWO/6CNOs compared with pure BWO. Besides, the samples show a higher discharge time at low current density, and the discharge time decreases with the increase in current density. Thus, the specific capacitances are calculated by the relation (Ng et al., 2009; Chen, 2013)

(9) $$\begin{array}{r} {\text{C}}_{\text{sp}}\;=\;\frac{2E}{m(V_{2}^{\;2}\;-\;V_{1}^{\;2})} \end{array}$$

where C_sp_ and *M* are the specific capacitance in GCD and the mass of active materials (kg). *E* is the energy density, which is obtained from the average slope of the discharge curve, and *V*_2_ and *V*_1_ are the maximum voltage (−0.2 V) and the minimum voltage (−1.1 V), respectively. The energy density E (in Wh/kg) and power density P (W/kg) at various current densities are calculated using the equations

(10) $$\begin{array}{r} \text{E }=\;\frac{I\;\int V\left(t\right)\text{dt}}{m} \end{array}$$

(11) $$\begin{array}{r} P\;=\;\frac{E}{t} \end{array}$$

Here, ∫*V*(*t*)*dt*, I, *m*, and t are the integral area of the discharge curves, the current density, the mass of active materials (kg), and the discharge time, respectively.

Herein, the calculated specific capacitances are presented in Figure 7C, and the values of BWO and BWO/6CNOs at a current density of 1 mA/cm^2^ are 533.2 and 955.8 F/g, while at a current density of 5 mA/cm^2^, the values are 271.8 and 500.8 F/g, respectively. It can be found that the specific capacitance of the two samples decreased with an increase in current density, which is caused increasing ionic resistivity and the charge not being able to diffuse into inner active sites (Senthilkumar et al., 2013). In addition, the decrease under a high current density is mainly due to the decrease at a higher voltage (Yuan et al., 2009). Furthermore, the calculated energy densities of BWO/6CNOs at 1 to 5 mA/cm^2^ are 46.5, 37.2, 29.7, 22.6, and 19.1 Wh/kg, respectively. This energy density decrease with an increase in the current density occurs mainly because the charges stored per unit mass/volume decline. In addition, the power density values of BWO/6CNOs are found to be 131.1, 247.4, 287.2, 357.2, and 550.2 W/kg for 1 to 5 mA/cm^2^. This increase with an increase in current density occurs because the charge/discharge rate per unit mass/volume increases.

To investigate the electrochemical cycle stability of the samples, repeat GCD studies were carried out. Figure 7D shows the capacitance retention at a current density of 3 mA/cm^2^ for 1,000 cycles. The initial discharge capacitance of BWO and BWO/6CNOs is observed to be 309.2 and 640.2 F/g, respectively, and the specific capacitance gradually decreases to 218.1 and 575.6 F/g, maintaining 80.5% and 90.1% of the initial capacitance. Thus, the BWO/6CNO composite shows better cycling stability than does pure BWO. Compared with Bi_2_O_3_ (304 F/g at a current density of 3 mA/cm^2^), BiPO_4_ (302 F/g at a current density of 2 mA/cm^2^), Bi_2_MoO_6_ (171.3 F/g at a current density of 0.585 A/g), and CuBi_2_O_4_ (297 F/g at a current density of 3 mA/cm^2^) (Vivier et al., 2001; Khan et al., 2014; Nithya et al., 2015; Ma Y. et al., 2016), the BWO/6CNO composite prepared in this manuscript shows excellent electrochemical properties, which indicates the practicability of the material.

**Figure 8:**
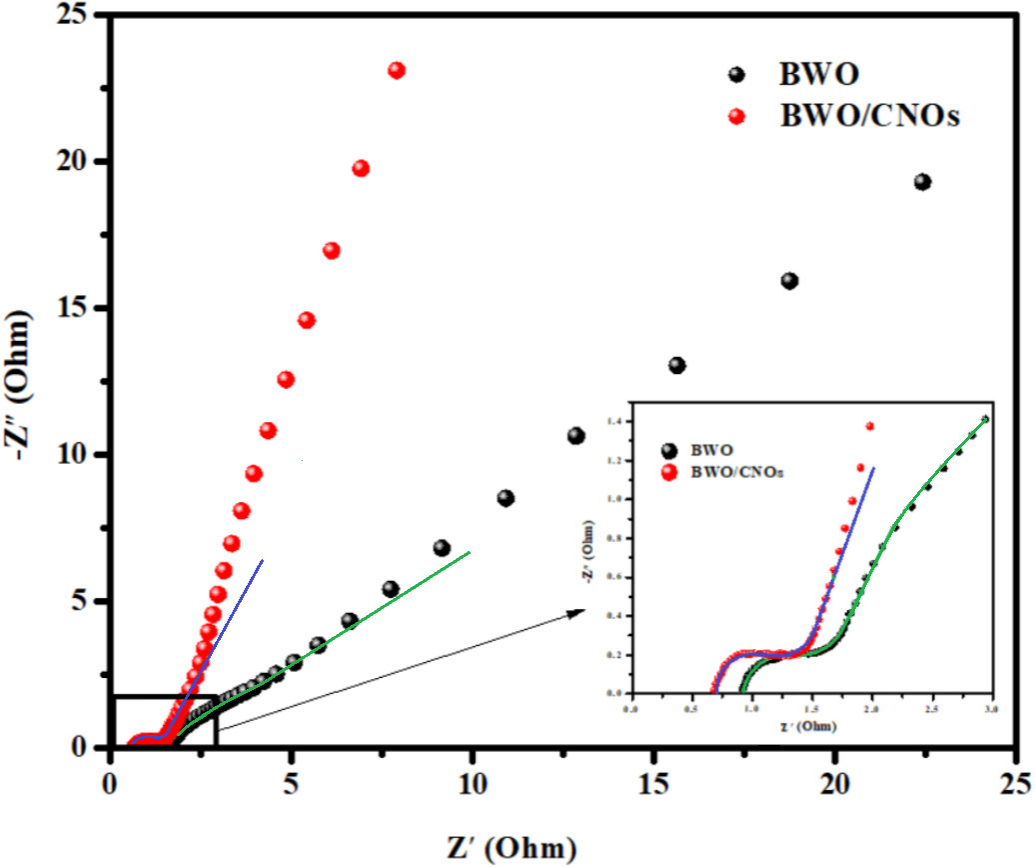
Nyquist spectra of BWO and BWO/6CNO composite, inset: the enlarged high-frequency region.

EIS measurements were carried out to examine the electrochemical properties of BWO and BWO/6CNOs. Nyquist spectra of the samples are shown in Figure 8, and the inset is an enlargement of the high-frequency region. As seen from Figure 8, both spectra are of similar type, i.e., a semicircle in the high-frequency region and a straight line in the low-frequency region, which is defined as Warburg impedance. The semicircle at high frequency corresponds to the resistance between the reference electrode and the working electrode (R_s_), and the smaller semicircle indicates the smaller charge-transfer resistance (R_ct_), which suggests that the Faradic reaction proceeds easily with high reversibility. The straight line is related to the diffusion of electrolyte ions into/from the pores of the electrode, and the steeper tail slope indicates a lower diffusion resistance. The semicircle in the high-frequency region is the parallel combination of charge transfer (R_ct_) and double-layer capacitance (C_dl_) (Yang S. H. et al., 2018). The EIS equivalent circuits of BWO and BWO/6CNOs were shown in Table 1. The equivalent circuit of BWO/6CNOs includes R_s_, C_dl_, R_ct_, and Warburg impedance, and the parameters are also presented in Table 1. Unlike BWO/6CNOs, the equivalent circuit of BWO includes two CPEs (constant phase elements), which are used to replace the pure capacitive element to get better data fitting. Also, R_f_ in BWO shows a passivation film on the surface of BWO, which gives evidence for the enhanced electrochemical performance of BWO/6CNOs. Moreover, the slope at low frequency stands for Warburg resistance and can reflect ideal diffusion resistance. In addition, the Nyquist plot of BWO/6CNOs in the low-frequency region shows an almost ideal straight line along the imaginary axis compared with that of pure BWO, which suggests that the composite has low diffusion resistance.

**Table 1:** Equivalent circuit impedance parameters of BWO and BWO/CNOs fitted from Nyquist plots.

**Impedance values**	**Equivalent circuit**	**Parameters**							
		**R_**s**_ (Ω)**	**R_**f**_ (Ω)**	**R_**ct**_ (Ω)**	**W (Ω/s^**1/2**^)**	**Y_**01**_ (**Ω^−1^** s^**-n**^)**	**n_**1**_**	**Y_**02**_ (**Ω^−1^** s^**-n**^)**	**n_**2**_**
BWO	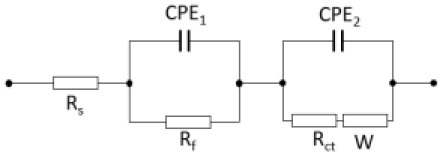	1.97	0.56	1.21	1.74	0.047	0.93	0.78	0.31
BWO/6CNOs	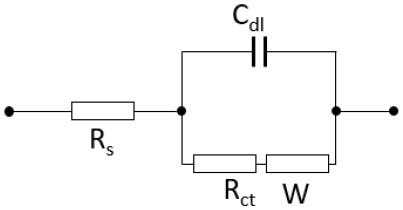	**R**_***s***_ **(**Ω**)** 1.53		**R**_**ct**_ **(**Ω**)** 0.79		**W (**Ω**/s**^**1/2**^**)** 1.51		**C**_***dl***_ **(F)** 2.74 × 10^−6^	

Based on the above analyses, some possible reasons for the excellent performance of BWO/6CNO composites can be concluded as follows. First, the well-dispersed nanoflake shape of BWO/6CNOs gives them a larger surface area and pore volume, which provide more chances for the active materials to accept more ions at the same time (Vivier et al., 2000). Second, CNOs in the composites act as a flexible matrix leading to the volume invariance during charging-discharging cycling, which gives excellent durability to the active materials according to the BET results. Third, it is worth mentioning that the excellent conductivity of CNOs also plays an important role in improving the conductivity of BWO/6CNO composites (Liddell et al., 2015). Overall, BWO/6CNO composite shows outstanding electrochemical performance.

## Conclusion

In summary, BWO/CNO composite, as an electrode for a supercapacitor, was successfully synthesized *via* a simple hydrothermal method. The microstructure and chemical composition were studied by SEM, TEM, BET, and XPS measurements, and the results show heterojunction formation between BWO and CNOs. Electrochemical tests proved that BWO/6CNOs possessed enhanced electrochemical performances with a specific capacitance of 640.2 F/g at a current density of 3 mA/cm^2^ and a 90.1% capacitance retention after 1,000 charge-discharge cycles, which was substantially better than pure BWO (359.1 F/g and 80.5% capacitance retention). The obtained BWO/6CNOs exhibited high specific capacitance, superior cycling stability, and outstanding rate capability. The improved electrochemical performance resulted from the CNOs in the composites being able to provide an appropriate microporous structure and excellent electronic transport path, which would enhance the electrochemical performance of supercapacitors. In summary, the low cost, environmentally friendliness, and excellent electrochemical properties of the BWO/6CNO composites make them promising for developing capacitors with high specific capacitance, low resistivity, and high stability.

## Data Availability Statement

All datasets generated for this study are included in the article/Supplementary Material.

## Author Contributions

WZ, JW, and LZ designed and engineered the samples. JW and WZ performed the experiments. WZ, JW, LP, CG, LP, SC, and LZ performed the data analysis. JW, CG, and LZ wrote the paper. All authors contributed to the theoretical analysis and the general discussion.

## Conflict of Interest

The authors declare that the research was conducted in the absence of any commercial or financial relationships that could be construed as a potential conflict of interest. The handling Editor declared a shared affiliation, though no other collaboration, with one of the authors JW.
