# Supplementary material for: High Electrochemical Performance of Bi2WO6/Carbon Nano-Onion Composites as Electrode Materials for Pseudocapacitors
Source: Front Chem. 2020 Jul 31;8:577. doi: 10.3389/fchem.2020.00577 (PMC7411300; doi:10.3389/fchem.2020.00577)
Supplement: Supplementary file 1 [file Image_1.pdf]

High Electrochemical Performance of Bi_2WO_6/Carbon Nano-Onion Composites as Electrode Materials for Pseudocapacitors

Weike Zhang [1*], Lin Peng [2], Jiawei Wang [3], Chunli Guo [4], Siew Hwa Chan [5], Lan Zhang [5*]

[1] Institute of New Carbon Materials, Taiyuan University of Technology, 79 West Yingze Street, Taiyuan, P. R. China

[2] Beijing Huaxin Zhiyuan Taiyuan Branch, 2 Xinghualing Street, Taiyuan, P. R. China

[3] School of Chemical Engineering and Technology, Tianjin University, Tianjin, P. R. China

[4] School of Material Science and Engineering, Taiyuan University of Technology, 79 West Yingze Street, Taiyuan, P. R. China

[5] Energy Research Institute at NTU (ERIAN), Nanyang Technological University, Singapore, Singapore

*** Correspondence:**

Weike Zhang

zhangweike@tyut.edu.cn

Lan Zhang

zhanglan@ntu.edu.sg

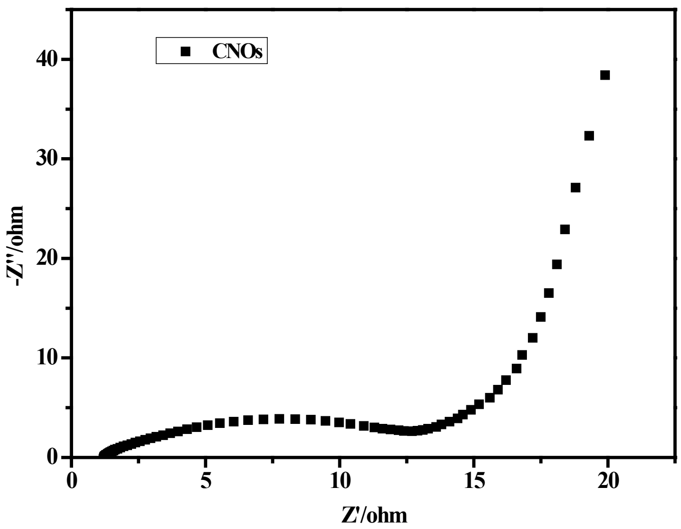

Fig. S1 Nyquist spectra of CNOs.
